# Supplementary material for: Non-genetic neuromodulation with graphene optoelectronic actuators for disease models, stem cell maturation, and biohybrid robotics
Source: Nat Commun. 2025 Aug 20;16:7499. doi: 10.1038/s41467-025-62637-6 (PMC12368249; doi:10.1038/s41467-025-62637-6)
Supplement: Supplementary file 1 — Supplementary Information [file 41467_2025_62637_MOESM1_ESM.pdf]

SUPPLEMENTARY INFORMATION

Non-Genetic Neuromodulation with Graphene Optoelectronic Actuators for Disease Models, Stem Cell Maturation, and Biohybrid Robotics

Elena Molokanova[1,2*], Teng Zhou[2,3], Pragna Vasupal[2], Volodymyr P. Cherkas[4,5], Prashant Narute[6], Mariana S.A. Ferraz[7,8], Michael Reiss[3], Angels Almenar-Queralt[7], Georgia Chaldaiopoulou[7], Janaina Sena de Souza[7], Honieh Hemati[3], Francisco Downey[1,3], Omowuyi O. Olajide[3], Carolina Thörn Perez[9], Francesca Puppo[7,10], Pinar Mesci[7†], Samuel L. Pfaff[9], Dmitry Kireev[6], Alysson R. Muotri[7,10,11*], Alex Savchenko[1*]

[1]Nanotools Bioscience, La Jolla, CA 92037, USA; [2]NeurANO Bioscience, La Jolla, CA 92037, USA; [3]Shu Chien-Gene Lay Department of Bioengineering, School of Engineering, University of California San Diego, La Jolla, CA, 92093, USA; [4]Institute of Bioorganic Chemistry, Polish Academy of Sciences, Poznan, Poland; [5]Bogomoletz Institute of Physiology, Kyiv, Ukraine; [6]Department of Biomedical Engineering, University of Massachusetts, Amherst, Amherst, MA, 01003, USA; [7]Department of Pediatrics, School of Medicine, University of California San Diego, La Jolla, CA, 92093, USA; [8]Neurogenetics Laboratory, Universidade Federal do ABC, São Bernardo do Campo, SP 09606-045, Brazil; [9]The Salk Institute for Biological Studies, La Jolla, CA, 92027, USA; [10]Department of Cellular and Molecular Medicine, School of Medicine, University of California San Diego, La Jolla, CA, 92093, USA; [11]Sanford Consortium for Regenerative Medicine, La Jolla, CA, 92037, USA

***Corresponding authors' contact info:**
Elena Molokanova, Ph.D., NeurANO Bioscience, La Jolla, CA 92037
E-mail address: emolokanova@neuranobio.com
Alysson R. Muotri, Ph.D., University of California San Diego, CA 92093
E-mail address: muotri@ucsd.edu
Alex Savchenko, Ph.D., Nanotools Bioscience, La Jolla, CA 92037
E-mail address: asavtchenko@nanotoolsbio.com

†Present address: Axiom Space, Houston, TX, 77058

Characterization of GraMOS Materials through Analytical Techniques

The schematic illustration comparing GO and rGO is shown in Supplementary Fig. 1a. Pristine graphene features largely intact hexagonal lattice, while GO is highly defect-rich and populated with oxygen-containing groups such as hydroxyls, epoxides, and carboxyls. As partially reduced, the rGO flakes still bear a number of oxygen and hydroxyl groups, making it highly hydrophilic, and allowing for tight contact with biological membranes.

Supplementary Fig. 1b shows the UV-Vis absorption spectra of rGO normalized to that of original GO. The spectrum of the GO suspension exhibits a strong absorption peak at 237 nm, corresponding to the $\pi \rightarrow \pi^*$ transition of C=C bonds, along with a shoulder peak at 300 nm, attributed to the $n \rightarrow \pi^*$ transition of C=O bonds[1]. Upon reduction of GO to rGO, a redshift in the maximum absorption peak to 268 nm is observed, together with disappearance of the $n \rightarrow \pi^*$ transition band at 300 nm and a significant increase in absorption in the near-infrared region (Fig. 2a). These observations indicate the successful reduction of GO to rGO, primarily due to the near-complete removal of oxygen-containing functional groups from the basal plane, restoration of the conjugated π-electron[2].

XRD was employed to verify the successful reduction of GO to rGO by analyzing changes in the crystal structure and interlayer spacing (Supplementary Fig. 1c). GO typically shows a characteristic XRD peak of 2*θ* of around 10-12°, corresponding to an interlayer spacing (*d*-spacing) of about 0.8-1.0 nm due to the presence of oxygen-containing functional groups[3]. Upon reduction to rGO, the oxygen-containing groups are removed, leading to a decrease in interlayer spacing, as a result a new peak of 2θ of 24-26°, corresponding to a *d*-spacing of about 0.34 nm. In our study, the presence of characteristic broad peak at around 2θ of 26°, corresponds to (002) plane, and absence of peak at around 12°, corresponds to (001) plane of the graphite (appears in GO), confirms the successful reduction of GO[4]. This is attributed to removal of carbonaceous oxygen moieties of GO in the reduction process, thus the decrease in inter-planner spacing in resulting rGO[4-8].

Raman spectroscopy was utilized to characterize the molecular composition and structure of our materials. Raman spectra of our materials (Supplementary Fig. 1d) show three major characteristic bands: (i) the G band at ~1595 cm^{-1}, which originates from the in-plane stretching of the graphene lattice and indicates the degree of graphitization; (ii) the D band at ~1330 cm^{-1}, associated with lattice distortions of carbon bonds, functionalization of the graphene plane, corrugations, folds, and dangling carbon bonds at the edges of rGO flakes; and (iii) the 2D band peak at ~ 2660 cm^{-1}, which is characteristic of sp^2 hybridization in graphitic materials[9]. The D band is especially sensitive to edge defects and domain sizes[10], and thus, the intensity ratio between the D and G peaks (I_D/I_G) is a useful measure for distinguishing between GO and rGO as reduction leads to the removal of oxygen moieties, resulting in smaller-sized domains in which sp^2 vibrations may occur. The I_D/I_G of ~1.03

suggests that rGO is functionalized, resulting in a higher proportion of sp^3 hybridized carbon atoms. Additionally, a consistent peak of D+D' at ~2930 cm^{-1} was observed. Appearance of these bands is related to the interaction of the incorporated amine moieties with rGO[4,11].

FTIR was employed to confirm the nitrogen content in the synthesized rGO, the FTIR spectra of rGO in Supplementary Fig. 1e highlights their nitrogen content. The spectrum features a peak at 1634 cm^{-1} (N-H deformation in primary amine), a broad peak at 3200 – 3500 cm^{-1} (-OH groups at 3495 cm^{-1} and N-H bonding of primary amine at 3276 cm^{-1}), and a peak at 2101 cm^{-1} (C-N stretching). These peaks confirm the amine functionalization of rGO[2,4,11,12]. Therefore, GO reduction in ammonia appears to lead to the incorporation of electron-donating elements (such as nitrogen) and an increase in the concentration of electrons (n-type doping).

To analyze the height and potential gradient across rGO flakes, the surface contact potential difference (CPD) was quantified using Atomic Force Microscopy (AFM) (Supplementary Fig. 2) and AM-KPFM imaging (Supplementary Fig. 3 a-b). For each rGO flake (Flake #1 and #2), the thickness and potential variation were determined by averaging five independent height and potential profiles. A representative height and potential profile are shown in Supplementary Fig. 3c and 3d, respectively. The average height of rGO flakes #1 and #2 was estimated of 1.47 ± 0.37 nm and 1.85 ± 0.32 nm, respectively, and the corresponding potential differences between the Si substrate and the rGO flakes of −63 ± 9 mV (flake #1) and −41 ± 7 mV (flake #2). The observed negative potential gradient suggests that electrons are trapped within the rGO flakes, resulting in localized negative charge accumulation. These results complement the outcomes of FTIR and Raman spectroscopy, suggesting that the rGO flakes are negatively charged.

Finally, we evaluated the photo-response characteristics of rGO flakes by fabricating the electrolyte-gated rGO-based field-effect transistor (FET) device on an interdigitated electrode (IDE) array (Supplementary Fig. 6a-c). The transfer characteristics exhibited a V-shaped curve typical of graphene-based devices (Supplementary Fig. 6c). Upon exposure to low-intensity UV light (1.8 mW/cm^2), we observed a shift in the charge neutrality point (CNP) to the right, by almost 100mV, which indicates light-induced doping and highlights the significant influence of UV light on the electronic properties of rGO driven by photo-induced charge carrier dynamics. Furthermore, the process is dynamic (Supplementary Fig. 6d): in this case, the transistor is sampled at an operational point, sampling the drain-source current over time while the laser is shone onto the transistor for fixed periods of time (~15 sec), resulting in immediate change of current – as expected by above-described model.

SUPPLEMENTARY FIGURES

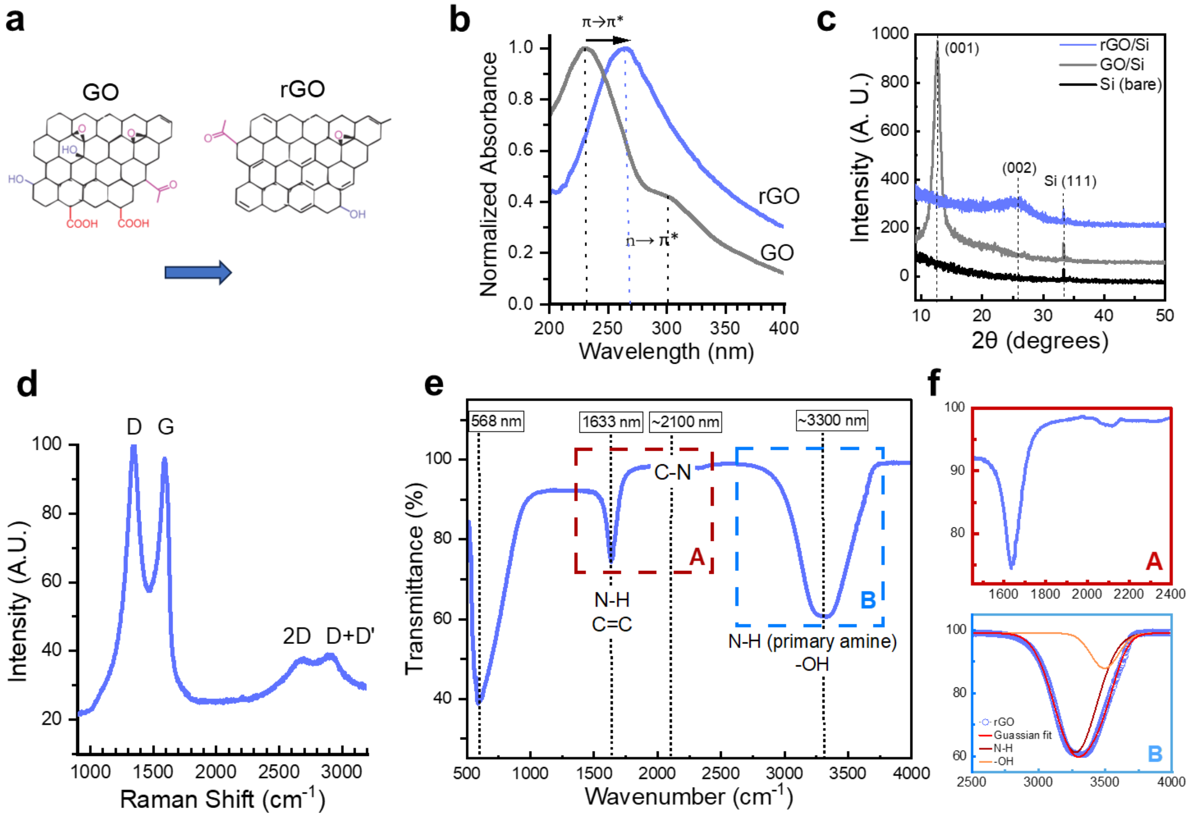

Supplementary Figure 1: Characterization of graphene substrates. a, Chemical conversion of GO into rGO. **b**, UV-Vis spectra of the initial GO (gray) and rGO (blue) after reduction. The peak at ~230 nm (GO) redshift to ~270 nm in rGO, disappearance of shoulder peak at 300 nm in rGO confirms successful synthesis of rGO. **c**, XRD pattern of rGO (blue) coated on Si wafer (grey). Peak at ~26° (2θ) confirms the successful synthesis of rGO. **d**, Raman spectra of rGO. **e**, Fourier Transform Infrared (FT-IR) spectrum of rGO with boxes A and B highlighting various nitrogen bandings in rGO. **f**, Top: Enlarged view of Box A shows peak at ~1633 nm corresponding to the N-H deformation in primary amine and C=C in rGO, whereas the peak at 2101 cm^{-1} corresponds to C-N stretching. X-Y axis labels as in (**e**) where the entire FT-IR spectrum is shown. **f**, Bottom: Enlarged view of Box B, where FTIR spectra was deconvoluted to separate the combination from different bonding, two components were identified; peaks at 3278 nm and 3494 nm are corresponding to N-H of primary amine and –OH bonding in rGO, respectively. The curve was fitted with a Gaussian function (R-square = 0.99782). X-Y axis labels as in (**e**) where the entire FT-IR spectrum is shown.

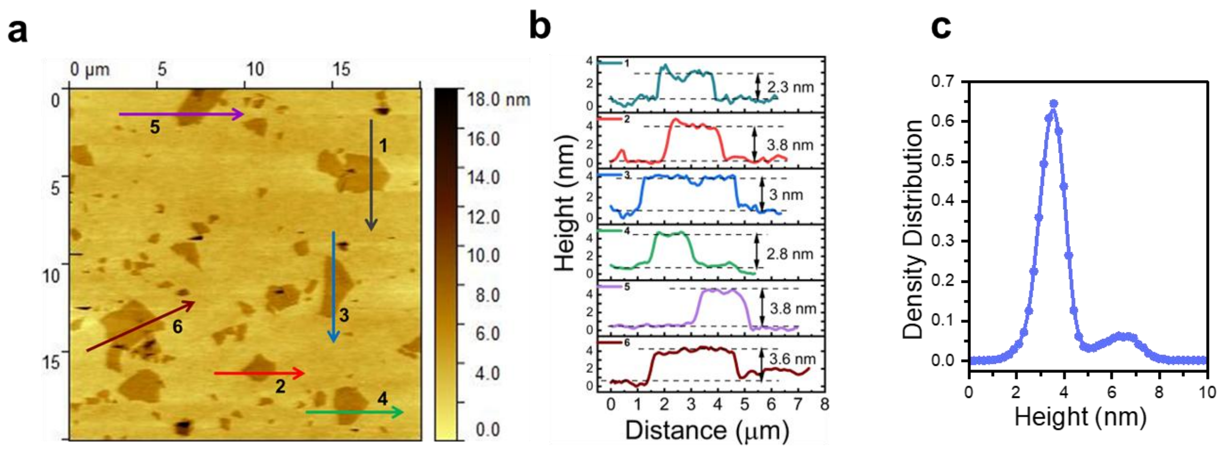

Supplementary Figure 2: Dimensions of rGO flakes. a, AFM image showing rGO flakes on the ITO-coated glass substrate. **b**, Height profiles of six rGO flakes (marked 1-6) shown in the AFM image in **(a)**. **c**, The histogram of the height measurements of rGO flakes.

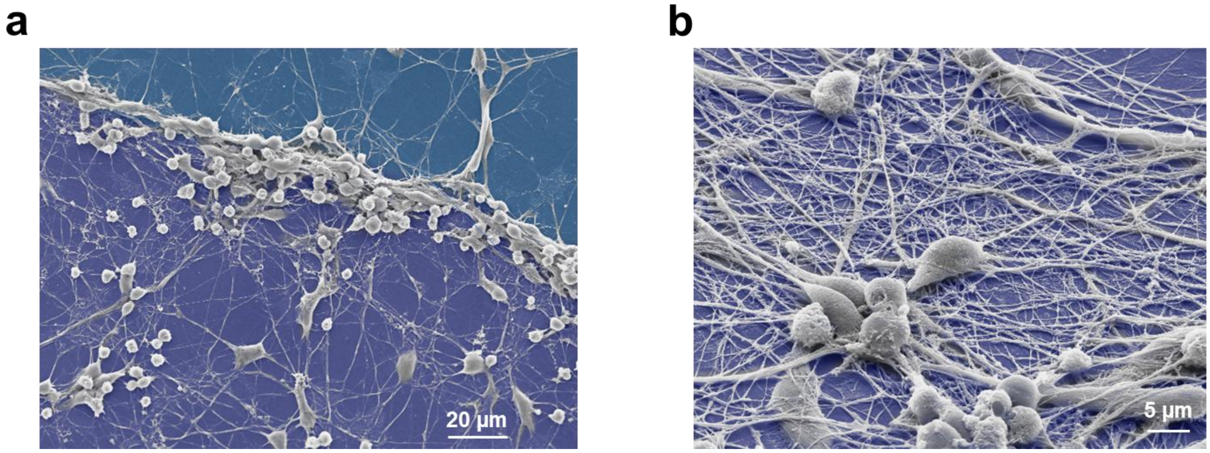

Supplementary Figure 3: **Scanning electron microscopy images of hiPSC-derived neurons on graphene** (selected from 97 images acquired across 3 differentiations)**. a**, hiPSC-derived neurons prefer G-coated (violet) over non-coated glass surfaces (blue). **b,** hiPSC-derived neurons form dense networks on G-coated surfaces (violet).

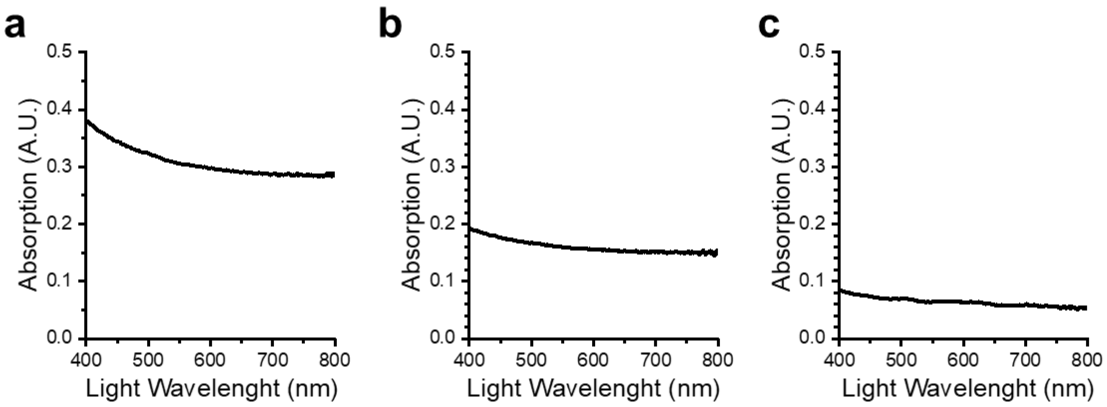

Supplementary Figure 4: Absorption spectra of G-coverslips with optical transmittance of approximately 50% (**a**), 70% (**b**), and 90% (**c**).

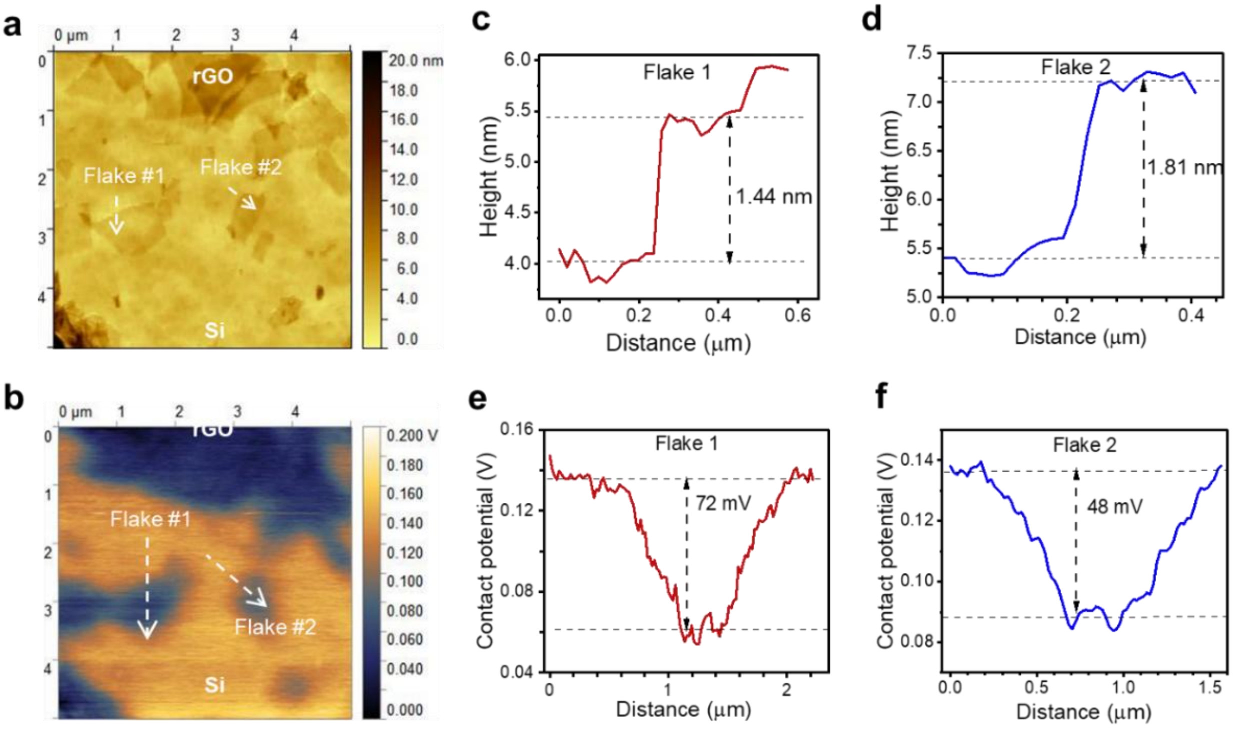

Supplementary Figure 5: Surface potential analysis of rGO. a, AFM topography image of rGO coated on the Si substrate and corresponding KPFM (**b**) image highlighting that rGO flakes bring additional negative potential. **c-d**, Representative height profiles of two of the flakes; **e-f**, Representative potential charge profile of the same two flakes measured along dashed lines in **(a)** and **(b)**, respectively.

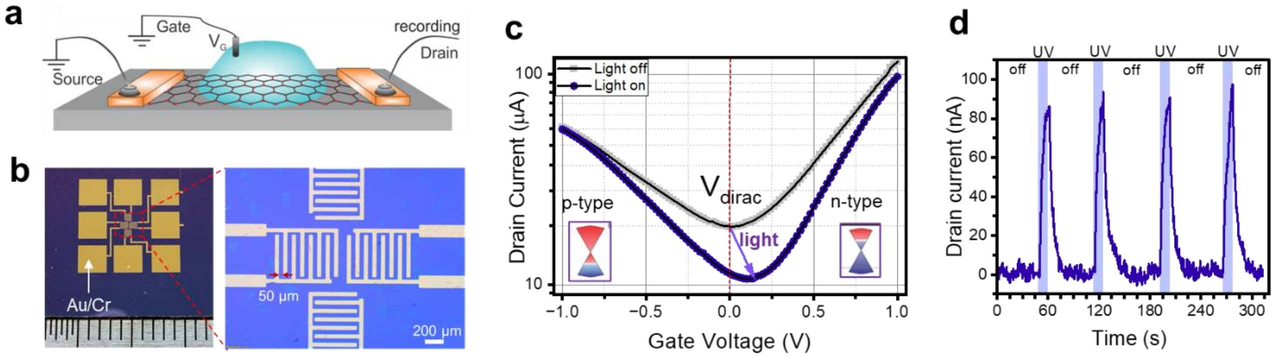

Supplementary Figure 6. Field-effect characteristics of the rGO. a, Schematics showing working of the rGO-based field-effect transistor (rGO-FET). **b,** Photograph (left) and optical image (right) of an interdigitated electrode (IDE). (**c**) Transfer I-V curves in the dark (black) and during UV light exposure (blue), highlighting the impact of UV illumination on the device's electronic properties. **d**, Dynamic photocurrent response of rGO-FET under regular UV exposure on/off cycles. The UV light used in the experiments had a radiant intensity of 1.8 mW/cm^2 (λ=365 nm).

SUPPLEMENTARY REFERENCES

1 Chen, J. *et al.* An improved Hummers method for eco-friendly synthesis of graphene oxide. *Carbon* **64** 225-229 (2013).

2 Rabchinskii, M. K. *et al.* From graphene oxide towards aminated graphene: Facile synthesis, its structure and electronic properties. *Scientific reports* **10** 6902 (2020).

3 Yang, Y. *et al.* Controllable reduction of graphene oxide by electron-beam irradiation. *RSC Advances* **9** 3597-3604 (2019).

4 Liu, R. *et al.* Nitrogen-functionalized reduced graphene oxide as carbocatalysts with enhanced activity for polyaromatic hydrocarbon hydrogenation. *Catal. Sci. Technol* **7** 1217-1226 (2017).

5 Kar, T. *et al.* Reduction of graphene oxide–a comprehensive electrochemical investigation in alkaline and acidic electrolytes. *RSC Advances* **4** 57781-57790 (2014).

6 Rajagopalan, B. & Chung, J. S. Reduced chemically modified graphene oxide for supercapacitor electrode. *Nanoscale research letters* **9** 1-10 (2014).

7 Li, H. *et al.* Enhanced cycle performance of Li/S battery with the reduced graphene oxide/activated carbon functional interlayer. *J. Energy Chem.* **26** 1276-1281 (2017).

8 Wang, C. *et al.* One-pot synthesis of N-doped graphene for metal-free advanced oxidation processes. *Carbon* **102** 279-287 (2016).

9 Bagri, A. *et al.* Structural evolution during the reduction of chemically derived graphene oxide. *Nature chemistry* **2** 581-587 (2010).

10 Stankovich, S. *et al.* Synthesis of graphene-based nanosheets via chemical reduction of exfoliated graphite oxide. *carbon* **45** 1558-1565 (2007).

11 Vincent, T. *et al.* Immobilization of metal hexacyanoferrates in chitin beads for cesium sorption: synthesis and characterization. *Journal of Materials Chemistry A* **2** 10007-10021 (2014).

12 Lee, A. Y. *et al.* Raman study of D* band in graphene oxide and its correlation with reduction. *Applied surface science* **536** 147990 (2021).
