# Supplementary material for: Non-genetic neuromodulation with graphene optoelectronic actuators for disease models, stem cell maturation, and biohybrid robotics
Source: Nat Commun. 2025 Aug 20;16:7499. doi: 10.1038/s41467-025-62637-6 (PMC12368249; doi:10.1038/s41467-025-62637-6)
Supplement: Supplementary file 2 — Description of Additional Supplementary Files [file 41467_2025_62637_MOESM2_ESM.pdf]

Description of Additional Supplementary Files

Supplementary Movie 1: Wide-field GraMOS-empowered all-optical calcium imaging in G-interfaced 3-month-old hiPSC-derived neurons.

Supplementary Movie 2: GraMOS-empowered all-optical calcium imaging in G-interfaced, 3-week-old hiPSC-derived neurons in response to a single, spatially constrained light pulse.

Supplementary Movie 3: Spatio-temporal activation maps of G-interfaced 3-week-old WATD and M233L hiPSC-derived neurons.

Supplementary Movie 4: A robotic system controlled by electrical signals from GraMOS-activated G-interfaced brain cortical organoids.
