# Supplementary material for: Non-genetic neuromodulation with graphene optoelectronic actuators for disease models, stem cell maturation, and biohybrid robotics
Source: Nat Commun. 2025 Aug 20;16:7499. doi: 10.1038/s41467-025-62637-6 (PMC12368249; doi:10.1038/s41467-025-62637-6)
Supplement: Supplementary file 7 — Reporting Summary [file 41467_2025_62637_MOESM7_ESM.pdf]

nature portfolio

Corresponding author(s): Elena Molokanova, Alysson R. Muotri, Alex Savchenko

Last updated by author(s): Jul 2, 2025

Reporting Summary

Nature Portfolio wishes to improve the reproducibility of the work that we publish. This form provides structure for consistency and transparency in reporting. For further information on Nature Portfolio policies, see our Editorial Policies and the Editorial Policy Checklist.

Statistics

For all statistical analyses, confirm that the following items are present in the figure legend, table legend, main text, or Methods section.

n/a	Confirmed	
☐	☒	The exact sample size (*n*) for each experimental group/condition, given as a discrete number and unit of measurement
☐	☒	A statement on whether measurements were taken from distinct samples or whether the same sample was measured repeatedly
☐	☒	The statistical test(s) used AND whether they are one- or two-sided<br>*Only common tests should be described solely by name; describe more complex techniques in the Methods section.*
☐	☒	A description of all covariates tested
☐	☒	A description of any assumptions or corrections, such as tests of normality and adjustment for multiple comparisons
☐	☒	A full description of the statistical parameters including central tendency (e.g. means) or other basic estimates (e.g. regression coefficient) AND variation (e.g. standard deviation) or associated estimates of uncertainty (e.g. confidence intervals)
☐	☒	For null hypothesis testing, the test statistic (e.g. *F*, *t*, *r*) with confidence intervals, effect sizes, degrees of freedom and *P* value noted<br>*Give P values as exact values whenever suitable.*
☒	☐	For Bayesian analysis, information on the choice of priors and Markov chain Monte Carlo settings
☒	☐	For hierarchical and complex designs, identification of the appropriate level for tests and full reporting of outcomes
☒	☐	Estimates of effect sizes (e.g. Cohen's *d*, Pearson's *r*), indicating how they were calculated

Our web collection on statistics for biologists contains articles on many of the points above.

Software and code

Policy information about availability of computer code

Data collection	pCLAMP 11 (Molecular Devices), Fusion Pro – Andor Technology (Oxford Instruments Group), ZEN 3 – Carl Zeiss Microscopy GmbH, Axis Navigator – Axion BioSystems
Data analysis	OriginPro 2025b – OriginLab Corporation (Northampton, MA, USA), Excel, Word – Microsoft Corporation (Redmond, WA, USA), Imaris – Oxford Instruments plc (Abingdon, UK), MATLAB – MathWorks Inc. (Natick, MA, USA), Python – Python Software Foundation, Fiji – Open-source;

For manuscripts utilizing custom algorithms or software that are central to the research but not yet described in published literature, software must be made available to editors and reviewers. We strongly encourage code deposition in a community repository (e.g. GitHub). See the Nature Portfolio guidelines for submitting code & software for further information.

Data

Policy information about availability of data

All manuscripts must include a data availability statement. This statement should provide the following information, where applicable:

- Accession codes, unique identifiers, or web links for publicly available datasets
- A description of any restrictions on data availability
- For clinical datasets or third party data, please ensure that the statement adheres to our policy

All data supporting the findings of this study are available within the article and its supplementary files. Any additional reasonable requests for information can be directed to the corresponding authors. Source data are provided with this paper.

Research involving human participants, their data, or biological material

Policy information about studies with human participants or human data. See also policy information about sex, gender (identity/presentation), and sexual orientation and race, ethnicity and racism.

Reporting on sex and gender	Not applicable
Reporting on race, ethnicity, or other socially relevant groupings	Not applicable
Population characteristics	Not applicable
Recruitment	Not applicable
Ethics oversight	Not applicable

Note that full information on the approval of the study protocol must also be provided in the manuscript.

Field-specific reporting

Please select the one below that is the best fit for your research. If you are not sure, read the appropriate sections before making your selection.

- [x] Life sciences
- [ ] Behavioural & social sciences
- [ ] Ecological, evolutionary & environmental sciences

For a reference copy of the document with all sections, see nature.com/documents/nr-reporting-summary-flat.pdf

Life sciences study design

All studies must disclose on these points even when the disclosure is negative.

Sample size	Sample sizes were chosen based on established practices in the field and previous experience with similar experimental designs involving graphene-based interfaces, electrophysiological recordings, and optical neuromodulation. In each case, the number of biological replicates was sufficient to ensure consistent, reproducible results across independent experiments, as reported in the corresponding figure legends.
Data exclusions	No excluded data
Replication	All key experiments were performed with appropriate biological replicates and independently repeated in accordance with Nature Communications' standards.
Randomization	Samples for immunohistochemistry (IHC) were randomized prior to staining and imaging to minimize bias. Randomization was applied to the assignment of samples to processing batches and imaging order. Data analysis was also performed blinded to experimental group identity where applicable.
Blinding	Blinding was consistently used throughout the project: e.g., during the analysis of data from fluorescent imaging studies, including cell viability assays, calcium imaging and immunohistochemistry (IHC) studies.<br>Blinding was not applicable to certain experiments where data acquisition and analysis relied on objective, instrument-based measurements without subjective interpretation.

Reporting for specific materials, systems and methods

We require information from authors about some types of materials, experimental systems and methods used in many studies. Here, indicate whether each material, system or method listed is relevant to your study. If you are not sure if a list item applies to your research, read the appropriate section before selecting a response.

Materials & experimental systems

n/a	Involved in the study
☐	☒ Antibodies
☒	☐ Eukaryotic cell lines
☒	☐ Palaeontology and archaeology
☒	☐ Animals and other organisms
☒	☐ Clinical data
☒	☐ Dual use research of concern
☒	☐ Plants

Methods

n/a	Involved in the study
☒	☐ ChIP-seq
☒	☐ Flow cytometry
☒	☐ MRI-based neuroimaging

Antibodies

Antibodies used

Primary antibodies used were: FOXG1 (Millipore, MABD79; 1:500), DCX (Abcam, ab18723; 1:200), MAP2 (Abcam, ab5392; 1:1000), ZO-1 (Invitrogen, 33-9100; 1:100), S-OPSIN (Invitrogen, OSR00219W; 1:500), RCVRN (Millipore, AB5585; 1:2000), CRX (RD Systems, AF7085; 1:100), RHO (Abcam, ab5417; 1:200), VGLUT1 (Synaptic Systems, 135311; 1:100), and GAD65+67 (Abcam, ab11070; 1:200). Secondary antibodies (Invitrogen; 1:500) used were: Goat anti-Rabbit Alexa Flour 488 (A11034), Donkey anti-Mouse Alex Flour 555 (A31553), Goat anti-Rabbit Alexa Flour 647 (A21244), Donkey anti-Mouse Alexa Flour (A21202), Donkey anti-Mouse Alexa Flour 647 (A31571), Donkey anti-Sheep Alexa Fluor 647 (A21448), and Goat anti-Chicken Alexa Flour 647 (A21449).

Validation

All antibodies are commercially available and fully validated by the antibodies' manufacturers. The corresponding citations and validation descriptions can be found on the manufacturers' websites.

Plants

Seed stocks

Not applicable

Novel plant genotypes

Not applicable

Authentication

Not applicable
